# Antineutrophil Cytoplasmic Antibody (ANCA)-Associated Vasculitis (AAV) Masquerading As Mononeuritis Multiplex: A Case Report

**DOI:** 10.7759/cureus.38651

**Published:** 2023-05-06

**Authors:** Anandkumar Patel, Umangkumar M Patel, Vani Sojitra

**Affiliations:** 1 Medicine, Maharshi Hospital Private Limited, Surendranagar, IND; 2 Neurology, Shalby Multispeciality Hospitals, Ahmedabad, IND; 3 Medicine, Bavadia Hospital, Una, IND

**Keywords:** anca associated vasculitis, granulomatosis with polyangiitis (gpa), cyclophosphamide methylprednisolone pulse, c-anca, mononeuritis multiplex

## Abstract

Antineutrophil cytoplasmic antibody (ANCA)-associated vasculitis (AAV) is a group of disorders that causes severe small-vessel inflammation with systemic manifestations. There are three subtypes of AAV, namely granulomatosis with polyangiitis (GPA), microscopic polyangiitis (MPA), and eosinophilic GPA (EGPA). The most commonly affected organs are the upper and lower respiratory tract and the kidneys with occasional and varied neurological manifestations. Here we report a case of a 61-year-old female who presented with a one-month history of numbness, paresthesia, and asymmetric distal weakness of both lower limbs without any bladder or bowel involvement. Similar complaints appeared in her upper limbs three days prior to admission. She also suffered from myalgia, arthralgia, reduced appetite, and lost 8-10 kg weight over the past six months. Her nerve conduction study (NCV) revealed asymmetrical, predominantly motor, mixed, axonal and demyelinating polyneuropathy affecting both lower limbs, which was suggestive of mononeuritis multiplex. After a detailed workup, she tested strongly positive for cytoplasmic ANCA (c-ANCA). Although there was no clinical involvement of the respiratory tract, a contrast-enhanced computed tomography scan of the thorax and abdomen showed multifocal subpleural and lung parenchymal soft tissue lesions and mediastinal and bilateral hilar lymphadenopathy suggestive of a granulomatous lesion. She was diagnosed with the GPA variant of ANCA-associated vasculitis. Remission induction was achieved with high-dose methylprednisolone and cyclophosphamide along with alternate-day cotrimoxazole. Remission was maintained with tapering doses of steroid and mycophenolate mofetil with a slow but sustained recovery. On follow-up after one year, she walked without support with mild residual burning paresthesia in both feet. This case highlights the fact that neurological symptoms can be the presenting manifestation of AAV, and clinicians should have a high level of suspicion for AAV in patients presenting with mononeuritis multiplex, especially after ruling out common causes. By considering such etiologies, it may be possible to diagnose the condition at an earlier stage and initiate treatment to prevent potential pulmonary or renal damage.

## Introduction

Mononeuritis multiplex can be the presenting manifestation of many diseases like Hansen’s disease, diabetes, amyloidosis, and various systemic vasculitis. Amongst all vasculitis, antineutrophil cytoplasmic antibody (ANCA)-associated vasculitis (AAV) is characterized by loss of tolerance to neutrophil primary granule proteins, leukocyte proteinase 3 (PR3) or myeloperoxidase (MPO), with subsequent development of autoantibodies [1]. They cause severe small vessel inflammation, leading to endothelial injury and tissue damage.

There are three AAVs, namely granulomatosis with polyangiitis (GPA) (formerly known as Wegener’s granulomatosis), microscopic polyangiitis (MPA), and eosinophilic GPA (EGPA) (formerly known as Churg-Strauss syndrome). Out of them, GPA is predominantly associated with PR3-ANCA (cytoplasmic ANCA (c-ANCA)) positivity, and it presents with characteristic involvement of the ear, nose, throat, lungs, and kidneys like recurrent rhinosinusitis, earache, cough, hemoptysis, dyspnea, hematuria, and proteinuria, with or without renal dysfunction. The nervous system can be involved in forms of mononeuritis multiplex, sensory neuropathy, cranial nerve abnormalities, sensorineural hearing loss, and central nervous system mass lesions [2,3]. Peripheral nervous system involvement is seen in up to 15% of patients with GPA and usually occurs after a prolonged course of sinusitis, lung manifestations, or renal involvement [4,5]. Rarely, it can be the presenting manifestation. Very few cases of GPA presenting with mononeuritis multiplex have been reported. Here, we add another such case to the existing literature.

## Case presentation

A 61-year-old right-handed female, homemaker, with no previous comorbidities, presented with a one-month history of numbness, paresthesia, and weakness of both lower limbs. It started with numbness and a burning sensation in the bottom of both feet, which was more severe in her right foot. After a few days, she also noticed weakness in the form of difficulty in wearing slippers, as she needed to take support from a wall to insert her feet and there was slippage of the slippers without her awareness. It gradually progressed to the point that she started tripping while walking, especially over the right side, which also led to one episode of a near fall. Similar complaints of paresthesia and weakness developed in both hands over the three days before admission. She felt burning in her fingertips and was unable to do fine activities like breaking a chapati, buttoning-unbuttoning, and tying a knot. She didn't have any bladder or bowel difficulties. For the past five months, she had also been experiencing muscle and joint pain. She had lost significant weight (about 8-10 kg) over the past five months. She denied any addiction or toxin exposure.

Her general examination was unremarkable. On neurological examination, foot drop was apparent bilaterally (right > left). In her right lower limb, dorsiflexion (-3/5), as well as plantar flexion (3/5), were weak with difficult eversion and inversion. Similar but less severe weakness was observed in her left lower limb, with 3/5 power for dorsiflexion and -4/5 power for plantar flexion. Weakness of the small muscles of the hands and feet was present. No atrophy, fasciculation, or skin discolouration was seen. Decreased sensation to touch, pain, and temperature were found in both lower limbs up to below the knees (right > left) and both hands up to the wrist, which was not in any nerve distribution. Superficial plantar reflexes were mute bilaterally. Deep tendon reflexes were normal in the upper limbs, reduced at the knee (+1/+1), and absent at the ankle. Higher mental function, cranial nerves, cerebellar, and autonomic system examination was normal. She walked with a high steppage gait, and the Romberg test was normal.

Routine investigations were done, which showed hypochromic microcytic anaemia (haemoglobin (Hb) 10.3 g/dL, mean corpuscular volume (MCV) 79.4 fL, mean corpuscular Hb (MCH) 25.4 pg)), raised C-reactive protein (CRP) (68 mg/L), high erythrocyte sedimentation rate (ESR) (66 mm/1 hr), thrombocytosis (5,26,000/cmm), trace protein in urine without active sediments, hypoproteinemia (total protein 6.1 g/dL, albumin 2.58 g/dL, globulin 3.52 g/dL), and normal renal function (serum creatinine 0.40 mg/dL, urea 23.1 mg/dL, sodium 137.5 mmol/L, potassium 4.25 mmol/L). Her nerve conduction velocity (NCV) study showed changes of asymmetrical, predominantly motor, mixed, axonal and demyelinating polyneuropathy affecting both lower limbs (right peroneal and tibial nerves - conduction block, left tibial and peroneal nerves - severe axonal changes, absent H reflexes and F-waves bilaterally in lower limbs) suggestive of mononeuritis multiplex (Tables 1, 2). Human immunodeficiency virus (HIV), hepatitis B, and hepatitis C screening were negative. Serum antinuclear antibody (ANA), rheumatoid factor (RF), angiotensin-converting enzyme (ACE), and female tumour marker screening (serum alpha-fetoprotein, cancer antigen (CA) 15-3, CA 19.9, CA 125, and carcinoembryonic antigen) were also negative. Later, her c-ANCA (PR3-ANCA) was found to be strongly positive (25.8 AU/ml) with negative p-ANCA (myeloperoxidase (MPO)-ANCA).

**Table 1:** Motor nerve conduction study APB: abductor pollicis brevis; ADM: abductor digiti minimi; EDB: extensor digitorum brevis; AH: abductor hallucis

Nerve/Sites	Muscle	Latency (ms)	Amplitude (mV)	Distance (mm)	Latency Difference (ms)	Velocity (m/s)
Right Median - APB						
Wrist	APB	2.66	6.8	70		
Elbow	APB	7.14	6.6	240	4.48	53,6
Right Ulnar - ADM						
Wrist	ADM	2.29	6.9	70		
Below Elbow	ADM	6.93	6.1	250	4.64	53.9
Right Peroneal - EDB						
Ankle	EDB	3.70	6.0	80		
Fibular head	EDB	12.03	0,5	300	8.33	36.0
Left Peroneal - EDB						
Ankle	EDB	3,70	2.0	80		
Fibular head	EDB	11.25	1.8	320	7.55	42.4
Right Tibial - AH						
Ankle	AH	5.21	1.1	80		
Popliteal fossa	AH	14.79	0.1	390	9.58	40.7
Left Tibial - AH						
Ankle	AH	4.11	0.5	80		
Popliteal fossa	AH	13,33	0.3	390	9,22	42.3

**Table 2:** Sensory nerve conduction study

Nerve/Sites	Recording Site	Onset Latency (ms)	Peak Latency (ms)	Amplitude (µV)	Distance (mm)	Onset Latency Difference (ms)	Onset velocity (m/s)
Right Median - Orthodromic (Digit II, Mid palm)							
Digit II	Wrist	2.19	2.66	33.3	130	2.19	59.4
Right Ulnar - Orthodromic, (Digit V, Mid palm)							
Digit V	Wrist	1.72	2.19	12.0	110	1.72	64.0
Right Sural - Ankle (Calf)							
Calf	Ankle	2.19	2.97	9.9	110	2.19	50.3
Left Sural - Ankle (Calf)							
Calf	Ankle	1.93	2.76	11.1	110	1.93	57.1

**Figure 1:**
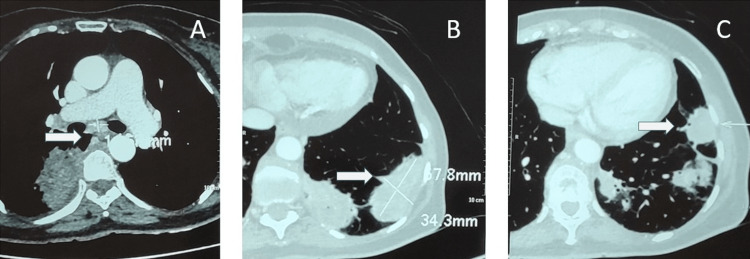
Mediastinal and bilateral hilar lymphadenopathy (A) along with multifocal subpleural and lung parenchymal soft tissue lesions in both lungs (B and C) suggesting the possibility of granulomatous etiology

Magnetic resonance imaging of the cervical spine with whole spine screening showed spondylotic changes at C4-C5 and C5-C6 levels. A contrast-enhanced computed tomography scan of the thorax and abdomen showed multifocal subpleural and lung parenchymal soft tissue lesions in both lungs and mediastinal and bilateral hilar lymphadenopathy, suggesting the possibility of a granulomatous etiology (Figure 1). The patient was advised for a biopsy, but she denied any invasive procedure.

Given the clinical presentation and findings of investigations, she was diagnosed with mononeuritis multiplex due to the GPA variant of AAV, according to the American College of Rheumatology (ACR) and the European Alliance of Associations for Rheumatology (EULAR), 2022 criteria. She was given high-dose methylprednisolone (1 g/day IV for five days) and cyclophosphamide (750 mg IV every two weeks for three doses and then every three weeks for three months) along with alternate-day cotrimoxazole. Remission was maintained with tapering doses of steroid and mycophenolate mofetil. She showed a slow but sustained recovery. On follow-up after one year, she could walk normally without any tripping, with mild residual burning paresthesia in both feet.

## Discussion

In 2012, the revised Chapel Hill consensus defined AAV as necrotizing vasculitis with few immune deposits or sometimes no immune deposits affecting small vessels, usually associated with p-ANCA or c-ANCA, but it can be ANCA negative too. Three major categories include GPA, MPA, and EGPA [6].

GPA is usually suspected in a patient who presents with clinical evidence of upper or lower respiratory tract involvement and/or glomerulonephritis along with constitutional symptoms. The suspicion greatly increases if there is laboratory detection of ANCA [7]. It most commonly occurs in older adults. Males and females are equally affected. Patients typically present with nonspecific symptoms like fever, anorexia, malaise, weight loss, arthralgias, and myalgias, which may last for weeks to months without evidence of specific organ involvement. As a result, GPA is frequently misdiagnosed initially as infections, malignancies, or inflammatory joint disease. When lesions involve specific systems, the clinical picture becomes clear. Ear, nose, and throat involvement is seen in 90% of cases in the form of nasal crusting, sinusitis, otitis media, earache, otorrhea, purulent or bloody nasal discharge, and oral or nasal ulcers. Conductive and/or sensorineural hearing loss is frequently seen, leading to severe permanent hearing impairment. Airways or pulmonary parenchyma involvement causes hoarseness, cough, dyspnea, stridor, wheezing, hemoptysis, or pleuritic pain. Renal involvement is also common in GPA, but very few patients have evident glomerulonephritis at presentation [8].

Neurological involvement is least common in GPA amongst all three AAVs and is seen as mononeuritis multiplex, sensory neuropathy, cranial nerve abnormalities, external ophthalmoplegia, sensorineural hearing loss, and central nervous system mass lesions. Peripheral nervous system involvement is seen in up to 15% of patients, with mononeuritis multiplex being the most common presentation. Commonly affected nerves include the peroneal, tibial, ulnar, and median nerves. This usually occurs after a prolonged course of sinusitis, lung manifestations, or renal involvement, but rarely it can also be the presenting manifestation, as seen in our case. Pathophysiologically, peripheral nervous system involvement is explained by the inflammation of the vasa nervorum, which leads to ischemia and subsequent axonal degeneration.

In 2022, the ACR and the EULAR developed and validated classification criteria for GPA using weighted parameters that are combined to derive a risk score. A total risk score of ≥5 is needed for the classification of GPA. Validation testing of these criteria has shown a sensitivity of 93% (95%CI 87% to 96%) and a specificity of 94% (95%CI 89% to 97%). As shown in Table 3, c-ANCA is a major discriminator within these criteria [9].

**Table 3:** ACR/EULAR 2022 classification criteria for granulomatosis with polyangiitis (GPA) ACR: American College of Rheumatology; EULAR: European Alliance of Associations for Rheumatology Sum the scores for 10 items, if present. A score of ≥5 is needed for the classification of granulomatosis with polyangiitis (GPA). Based on: Robson et al., 2022 [9]

Favouring		Non-favouring	
Positive test for cytoplasmic antineutrophil cytoplasmic antibodies (c-ANCA) or anti-proteinase 3 (anti-PR3) antibodies	+5	Blood eosinophil count - 1 x10^9^/liter	-4
Nasal involvement: bloody discharge, ulcers, crusting, congestion, blockage, or septal defect/perforation	+3		
Cartilaginous involvement (inflammation of ear or nose cartilage, hoarse voice or stridor, endobronchial involvement, or saddle nose deformity)	+2		
Pulmonary nodules, mass, or cavitation on chest imaging	+2	Positive test for perinuclear antineutrophil cytoplasmic antibodies (p-ANCA) or anti-myeloperoxidase (anti-MPO) antibodies	-1
Granuloma, extravascular granulomatous inflammation, or giant cells on biopsy	+2		
Inflammation, consolidation, or effusion of the nasal/paranasal sinuses, or mastoiditis on imaging	+1		
Pauci-immune glomerulonephritis on biopsy	+1		
Conductive or sensorineural hearing loss	+1		

Our patient had a positive test for c-ANCA and multifocal subpleural and parenchymal soft tissue masses in both lungs on chest imaging, which made the sum score of 7 favouring the diagnosis of GPA. Other differentials for mononeuritis multiplex like diabetes mellitus, retroviral infection, inflammatory demyelinating polyneuropathy, multiple nerve entrapments, and other vasculitides like sarcoidosis, lupus, and rheumatoid arthritis were already ruled out. Though not done in our case, the diagnosis of GPA should always be confirmed by a biopsy of the site of suspected active disease whenever possible.

There are only a few case reports of mononeuritis multiplex as a presenting manifestation of GPA. George et al. [10] and Aftab et al. [11] reported two similar cases of GPA but with additional renal dysfunction occurring during the course of the disease. Peshin et al. [12] and Lew et al. [13] also reported two such cases, but they had evidence of vasculitis on examination in the form of nailbed changes and Raynaud's phenomenon, respectively. Apart from these, there is one case report in German literature and one in a Portuguese journal on mononeuritis multiplex as the initial manifestation of GPA [14,15].

The presence of nervous system involvement has been shown to be a poor prognostic marker in patients with AAV. Hence, intensive therapy aiming to achieve rapid remission is desired at the earliest in such cases, which is followed by a maintenance phase intending to extend the remission and prevent a relapse. Though there isn't any universal protocol for dose and duration, an induction regimen consisting of high-dose glucocorticoids in combination with either rituximab or cyclophosphamide is commonly used to achieve remission, which is maintained using either rituximab, azathioprine, methotrexate, or mycophenolate mofetil [16]. Neuropathic pain can be managed symptomatically using tricyclic antidepressants (i.e., amitriptyline, nortriptyline), serotonin-norepinephrine reuptake inhibitors (i.e., duloxetine, venlafaxine), or antiepileptic drugs such as gabapentin and pregabalin.

## Conclusions

AAV is a rare disease entity with a varying presentation. Amongst all AAVs, neurological involvement is least common in GPA, but still, it can be the presenting manifestation, as seen in our case. Hence, in all cases of mononeuritis multiplex, especially when accompanied by the constitutional symptoms of a chronic inflammatory condition, a high index of suspicion should be kept for possible underlying AAVs after common causes have been ruled out. Immunosuppressive therapy with glucocorticoids plus cyclophosphamide can dramatically change the outcome of AAV, which otherwise carries a high mortality.
